# TB treatment initiation and adherence in a South African community influenced more by perceptions than by knowledge of tuberculosis

**DOI:** 10.1186/1471-2458-10-72

**Published:** 2010-02-17

**Authors:** Jane M Cramm, Harry JM Finkenflügel, Valerie Møller, Anna P Nieboer

**Affiliations:** 1 Erasmus University Rotterdam, institute of Health Policy & Management (iBMG), Rotterdam, The Netherlands; 2 Rhodes University, Institute of Social and Economic Research (ISER), Grahamstown, South Africa

## Abstract

**Background:**

Tuberculosis (TB) is a global health concern. Inadequate case finding and case holding has been cited as major barrier to the control of TB. The TB literature is written almost entirely from a biomedical perspective, while recent studies show that it is imperative to understand lay perception to determine why people seek treatment and may stop taking treatment. The Eastern Cape is known as a province with high TB incidence, prevalence and with one of the worst cure rates of South Africa. Its inhabitants can be considered lay experts when it comes to TB. Therefore, we investigated knowledge, perceptions of (access to) TB treatment and adherence to treatment among an Eastern Cape population.

**Methods:**

An area-stratified sampling design was applied. A total of 1020 households were selected randomly in proportion to the total number of households in each neighbourhood.

**Results:**

TB knowledge can be considered fairly good among this community. Respondents' perceptions suggest that stigma may influence TB patients' decision in health seeking behavior and adherence to TB treatment. A full 95% of those interviewed believe people with TB tend to hide their TB status out of fear of what others may say. Regression analyses revealed that in this population young and old, men and women and the lower and higher educated share the same attitudes and perceptions. Our findings are therefore likely to reflect the actual situation of TB patients in this population.

**Conclusions:**

The lay experts' perceptions suggests that stigma appears to effect case holding and case finding. Future interventions should be directed at improving attitudes and perceptions to potentially reduce stigma. This requires a patient-centered approach to empower TB patients and active involvement in the development and implementation of stigma reduction programs.

## Background

Tuberculosis (TB) is a global health concern. It is a major cause of illness and death worldwide, especially in low- and middle-income countries where it is fuelled by human immunodeficiency virus/acquired immune deficiency syndrome (HIV/AIDS), by population increase where TB is most prevalent and by increased poverty [1].

TB is the most common infection for the estimated 5.5 million South Africans living with HIV/AIDS (in a national population of some 48 million). The co-infection rate of HIV is estimated at 73 per cent in all TB cases. The estimated incidence of TB in South Africa is 692 per 100,000 people [2], a rate the WHO classifies as a serious epidemic. Even though the DOTS program has been active since 1995, TB remains a major health problem in South Africa and especially in the Eastern Cape [3]. Cure rate of 65% remains well below the 85% rate recommended by the WHO [2]. At 41%, The Eastern Cape's cure rate lags even further behind the national average [4].

Health seeking behaviour and non-adherence to therapy has been cited as major barrier to the control of TB [5-18]. Non-adherence is a complex, dynamic phenomenon with a wide range of interacting factors impacting treatment taking behaviour [10]. It poses a significant threat to both the individual patient and public health and is associated with higher transmission rates, morbidity, and costs of TB control programs [6]. Furthermore, it leads to persistence and resurgence of TB and is regarded as a major cause of relapse and drug resistance [7].

The TB literature is written almost entirely from a biomedical perspective. Recent studies show, however, that lay perceptions may explain why people seek and may stop taking treatment [10]. Research that acknowledges social, economic and geographical context is necessary to understand the impact of traditional beliefs and perceptions of illness and wellness on adherence [11].

Such research is still scarce. From the perspective of public health promotion, (cost-) effective treatment provision, individual wellbeing, and social welfare it is important to gain insight into the views of people regarding TB, especially among high risk populations such as the Eastern Cape [19,20]. Achieving a high level of TB awareness is crucial for the success of prevention and treatment efforts in high risk populations and represents a key challenge for public health initiatives [20]. Since the Eastern Cape is known as a province with high incidence, prevalence [21] and with one of the worst cure rates of South Africa [4], its inhabitants can be considered lay experts when it comes to TB. Therefore, we investigated perceptions among an Eastern Cape population. The aim of this study was to gain better insight into what influences health seeking behavior and adherence to TB treatment via investigating knowledge and perceptions regarding TB patients, health seeking behavior and adherence to TB treatment among an Eastern Cape community.

## Methods

The Eastern Cape is a South African province characterised by high levels of poverty and unemployment. TB is endemic in the Eastern Cape [2,21] and the cure rate of 41% lags far behind the 85% rate recommended by the WHO [4]. Data from a survey of 1020 Grahamstown East/Rhini households, conducted in November 2007, were used for this study.

### Ethical approval

The Rhodes University Ethical Standards Committee approved this research project. All personal identifiers have been removed or disguised so the person(s) described are not identifiable and cannot be identified through the details of the story.

### Sample design

An area-stratified sampling design was applied. Households were selected randomly in proportion to the total number of households in each neighbourhood. Within each of the 23 neighbourhoods, a random starting point was selected. Moving systematically through the neighbourhood, every tenth household was selected for inclusion. This method ensured that all households in all areas of Rhini stood an equal chance of being included in the survey. In each target household eligible respondents were identified. Eligibility criteria included adults over the age of 18 years with 6 months or more of residence in Rhini in the past year. One respondent per household was then selected using a Kish grid. The Kish grid provides a selection procedure by which eligible persons within the household stood an equal chance of being included in the survey [22]. If present at the time, the person selected was then interviewed. If the person was not available, arrangements were made to conduct the interview at a later time. Up to four visits were made to the household to interview the respondent.

Administering questionnaires among households was carried out by Development Research Africa, a well-known organization experienced in undertaking national probability based samples in deep rural and urban areas. With few exceptions, all questions were closed-ended items for which a set of response options was supplied. The full list of options is given in the respective tables.

### Instruments

Results from a focus group discussion held in 2006 with people living in the community under study identified several beliefs, perceptions and attitudes toward TB, people with TB, adherence with TB treatment, health seeking behavior and TB treatment types [21]. It appeared that the inhabitants are well informed on TB, Multi-Drug Resistant TB (MDR-TB) and Extensively-Drug Resistant TB (XDR-TB). Since numbers of MDR-TB and XDR-TB are increasing and these types require more extensive and toxic treatment, three questions related to knowledge on these TB types were included. The first question involves knowledge on TB in general; "TB can easily be cured if you take the right treatment". The second, "If you have MDR-TB it takes many months to be cured" assessed knowledge on MDR-TB. The third question " There is no cure at present for extremely drug resistant TB" concerned knowledge on XDR-TB, instigated by recent findings that XDR-TB can be treated through the use of aggressive regimens [1]. During the interview it was made clear to respondents which question addressed TB, MDR-TB and MDR-TB, respectively. Results from the focus groups reveal that there seems to be some misunderstanding about TB and the relation with HIV. Some people believe all TB patients will also develop HIV. Others mention TB to be a typical African disease and think that only poor people may get infected with TB [21]. We incorporated these issues in the TB knowledge test. In total, eight agree/disagree questions were presented to the respondent (correct = score 1, incorrect = score 0). The total score therefore ranges from 0 to 8, with higher scores indicative of greater knowledge.

The focus group results also revealed specific **perceptions and attitudes **toward TB within this community. People think that irresponsible individuals who do not take their treatment are mainly to blame for spreading TB. Besides blaming those individuals, they accuse them of hiding their TB status for fear of what others might say. They also think that people who acquire TB through drinking and smoking are getting what they deserve and that TB patients are less respected within the community [21]. This suggests that people might be subjected to a high level of stigmatization. These items were included in the questionnaire to investigate the extent to which people in this community share these attitudes and perceptions.

### Data analysis

Statistical analyses of attitudes and perceptions toward TB, adherence to TB treatment, health seeking behavior and TB treatment types consisted of frequency counts and percentages. Regression analysis and logistic regression analysis were performed to test whether differences in age, gender and education level led to different knowledge scores and different attitudes and preferences toward TB, adherence to TB treatment, health seeking behavior and TB treatment types. We performed listwise deletion of missing cases in the regression analyses. All statistical analyses were performed using SPSS 16.0.

## Results

### Respondent profile

An interview was obtained in 97.9% of the 1042 households targeted. Main reasons for not achieving an interview included non-availability of the respondent after four visits to the household, inability to give an interview for age or health reasons, and refusal due to disinterest or unwillingness.

In the 1020 households included in the final sample the majority (73%) of respondents were women. The median age of respondents was 38 years. Forty percent had completed some secondary education and 18% had matriculated. Approximately 7% had received post-matriculation education and training. Only 8% had no formal schooling.

These 1020 households counted 4245 persons. Just over a quarter (26%) were under 14 years of age. Some 43% were in the 15-59 years age group and 9.6% were over 60 years. The mean age was 30 years. The households included more women (56%) than men (44%) [21]. A detailed description of the study population can be found in the Institute of Social and Economic Research, Research Report Series No. 14 & No. 15 [23,24].

### Knowledge

The mean score on the TB knowledge test is 5.66 (with 8 being the highest score). 54% of the respondents believe TB to be an African disease and 60% believe that all TB patients will also develop HIV (Table 1). Most respondents correctly answered the other items. The score on the efficacy of treatment item "TB can easily be cured these days if you take your treatment" was high; 98% of the respondents gave the correct answer.

**Table 1:** Knowledge of TB

Knowledge items	Correct score	
	
	Frequency	Percent
TB is an African disease	469	46%
Only people who live in poverty get infected with TB	907	89%
Only people who are HIV positive get TB	907	89%
Anyone can get infected with TB because the germs are in the air	938	92%
TB can easily be cured these days if you take your treatment	1000	98%
If you have multi-drug resistant TB, it takes many months to be cured	836	82%
There is no cure at present for extremely drug resistant TB	643	63%
All people with TB develop HIV/AIDS	408	40%

### Lay experts' perceptions

To comprehend lay conceptualisations it is important to know the community's attitudes toward TB. Table 2 reflects this information in the studied population. Results show that 95% believe people with TB tend to hide their TB status because they are afraid of what others may say, 90% believe it is mainly the irresponsible patients who do not take their treatment that are to blame for spreading TB, 74% believes that people who get TB through drinking or smoking get what they deserve and 51% believe that if you have TB people do not respect you.

**Table 2:** Attitudes toward TB

	Frequency	Percent
Respondent believes people tend to hide when they have TB because they are afraid of what people say about them	967	95%
Respondent believes it is mainly irresponsible people who do not take their treatment who are to blame for spreading TB	918	90%
Respondent believes that people who get TB through drinking or smoking get what they deserve	724	71%
Respondent believes that if you have TB people do not respect you	520	51%

Table 3 shows the perceptions of respondents regarding (non) adherence to TB treatment. Thirty-two percent believe "they stick to the rules of treatment" is the most important thing that helps TB patients stay on treatment for 6 months, followed by "they want to show others that TB is like any other curable disease" (16%) and "they don't drink or smoke while on treatment" (15%).

**Table 3:** Perceptions toward adherence

Adherence items		Frequency	Percent
Most important thing that helps TB patients stay on treatment for 6 months	They stick to the rules of treatment	323	31.8
	They want to show others that TB is like any other curable disease	159	15.6
	They don't drink or smoke while on treatment	147	14.5
	They want to be cured so they can get on with their lives	124	12.2
	They have a sympathetic DOTS volunteer who helps them stay on treatment	87	8.6
	They have support from family and friends	72	7.1
	They have enough food to eat	43	4.2
	They don't listen when people gossip about them	38	3.7
	They are strong willed	23	2.3
	**Total**	**1016**	**100**
			
First main reason TB patients stop taking their treatment before they are cured	They feel better and think they are cured	547	53.8
	They are afraid people will talk badly about them when they go to the clinic to collect their pills	289	28.4
	They do not want to be seen standing in the same queue as people collecting Antiretroviral drugs (ARVs)	94	9.2
	They forget to take their medicine because they drink and smoke	64	6.3
	They are irresponsible and cannot be bothered to take their medicine	21	2.1
	Their families and friends are not supportive	2	0.2
	**Total**	**1017**	**100**
			
Second main reason TB patients stop taking their treatment before they are cured	They forget to take their medicine because they drink and smoke	414	41.2
	They are irresponsible and cannot be bothered to take their medicine	244	24.3
	They do not want to be seen standing in the same queue as people collecting ARVs	128	12.7
	Their families and friends are not supportive	122	12.1
	They are afraid people will talk badly about them when they go to the clinic to collect their pills	75	7.5
	They feel better and think they are cured	22	2.2
	**Total**	**1005**	**100**

As first main reason why TB patients stop taking their treatment before they are cured, 54% of the respondents feel this is caused by "patients feel better and think they are cured", followed by "they are afraid people will talk badly about them when they go to the clinic to collect their pills" (28%). As the second main reason, respondents believe this is due to "they forget to take their medicine because they drink and smoke" (41%), followed by "they are irresponsible and cannot be bothered to take their medicine" (24%).

The perceptions regarding TB treatment seeking behaviors are presented in Table 4. As most important reason why some people delay going to the clinic, 63% of respondents believe that is because "they are afraid they will be told they are HIV positive", followed by "they are afraid that people will talk about their visit to the clinic" (12%). The locations where respondents think TB patients prefer to take their daily treatment are the TB hospital (39%) and the clinic (33%).

**Table 4:** Perceptions toward treatment

TB treatment items		Frequency	Percent
Most important reason why some people delay going to the clinic	They are afraid they will be told they are HIV positive	640	63.0
	They are afraid that people will talk about their visit to the clinic	124	12.2
	They don't want to cough into the specimen bottle	64	6.3
	They are afraid that TB treatment will interfere with their social lives	64	6.3
	There are long queues at the clinics	40	3.9
	They are afraid they will lose their job/income	35	3.4
	They are afraid that TB treatment will be unpleasant and difficult	29	2.9
	They first wish to consult a traditional healer	20	2.0
	**Total**	**1016**	**100**
			
Location where most TB patients prefer to take their daily treatment	In the TB hospital where the nurses care of them	400	39.4
	In the clinic - they visit the clinic every morning to take their medicine	339	33.4
	At home and a DOTS volunteer visits them every day to bring the medicine	167	16.5
	At home/work and a family collects their medicine for them	108	10.7
	**Total**	**1014**	**100**

Regression analyses were performed to test for different knowledge scores and different attitudes and preferences toward people with TB, TB treatment adherence, case finding and TB treatment types among men and women, young and old, lower and higher educated. We found very few differences in outcomes and do not present these (tabulated information is available on request). Regression analysis of the TB knowledge test showed no significant differences in age, gender and education. Logistic regression analysis of age, gender and education with attitudes toward people with TB, perceptions regarding first and second main reason why TB patients stop taking their treatment before they are cured and beliefs of the population on TB treatment preferences of TB patients also showed no significant differences between respondents. Logistic regression analyses of perception of the lay public regarding the most important thing that helps TB patients stay on treatment for 6 months, showed that men and women slightly disagree on the item that "they stick to the rules of treatment" is the most important reason for TB patients to stay on treatment (mentioned less by women). Younger people more often mention "they have support from family and friends" than do the older respondents. As to the most important reason why some people delay going to the clinic, the higher educated more often mention "there are long queues at the clinics" and slightly more women mention "they are afraid people will talk about their visit to the clinic".

## Discussion

Regression analyses revealed similar attitudes and perceptions for the young and the old, for men and women, and for the lower and higher educated -- suggesting that people in this population share the same attitudes and perceptions. Our findings are likely to reflect the actual situation of TB patients in this population. The more so because most respondents know people with TB in their immediate environment.

TB knowledge can be considered fairly good among this community. The perception on "respondent believes it is mainly irresponsible people who do not take their treatment who are to blame for spreading TB" did not imply stigma only. Since some level of the treatment failure is due to irresponsible people this is also an indicator of knowledge.

However, respondents mainly considered TB to be an African disease and were under the misconception that all TB patients will also develop HIV. This perceived link can be explained by the fact that TB is main cause of death among the estimated 5.5 million South Africans living with HIV/AIDS (>10% of the country's population). Indeed, the co-infection rate approaches 73% in TB patients among all age groups [2]. Future publicly released information should emphasize that having active TB disease in an individual who is also infected with HIV leads to a worsening of the HIV illness, but having TB does not lead to becoming infected with HIV. Scores on the other items were fairly good, which contradicts prior findings suggesting that people in developing countries lack TB knowledge [8,16,19,20,25-27]. The population in the current study appears to be better informed in this regard.

The results are suggestive of a high level of stigmatization: a full 95% of respondents believe people with TB tend to hide their TB status because they are afraid of what others may say. People think that irresponsible individuals who do not take their treatment are mainly to blame for spreading TB. Besides blaming those individuals, they accuse them of hiding their TB status for fear of what others might say. They also think that people who acquire TB through drinking and smoking are getting what they deserve and that TB patients are less respected within the community. While research shows that the increased TB incidence and prevalence during the last decade is mainly due to population increase where TB is most prevalent, increase of HIV and increased poverty [28] most people within this community believe it is mainly the irresponsible patients who do not take their treatment that are to blame for spreading TB. Also the finding that respondents believe people who drink and smoke "get what they deserve" indicates blame and potential stigmatization.

More than half of the respondents believe that TB patients interrupt treatment because they think they are cured. This finding is interesting and justifies the need for improved communication and mutual understanding between care providers and patients. Implementing of interventions aimed at improving communication and mutual understanding between care providers and patients into this matter by the national tuberculosis program would improve TB treatment outcomes nationally. The second most important perception of respondents as main reason for non-adherence (they are afraid people will talk bad about them when they go to the clinic) is suggestive of stigma within the community. The fact that respondents mention this in their top 2 of most important reasons for default from treatment shows that people within this community believe stigma to be a real problem for adherence to TB treatment. This is in line with prior findings indicating that stigma plays a significant role in adherence [7,8,29-33]. If the perceptions of the respondents represent the actual situation for TB patients in the Eastern Cape, stigma causes people to reject the diagnosis, leading to the infection of more people and potential drug resistance.

Furthermore, it is interesting that most respondents believe TB patients prefer active involvement by travelling to the hospital and clinics themselves, compared to having a family member or DOTS volunteer daily delivering the medicine at home.

The major limitation of our study is that we studied perception of the lay public only, not actual behavior. Since attitudes of the lay public play a central role in the patient's decision-making process it is important to gain insight into what actually influences an individual's decision to seek treatment and adherence to that treatment instead of looking at behavior only. However, after learning how these mechanisms work it is important to investigate whether changes in attitudes and perceptions also lead to actual behavior change. Therefore, it would be interesting to conduct a follow-up study amongst TB suspects, adherent and non-adherent TB patients to document actual behavior.

The reason why the majority of respondents were women and older people were overrepresented in comparison to general population figures could be related to the inherent limitation of the conventional Kish grid [22]. Use of this instrument often leads to a higher proportion of women and older people. Regression analyses of age, gender and education show that there are very few significant differences in knowledge, attitudes and perception among these subgroups. Therefore, the higher proportion of women and older people does not appear to have affected our study findings.

## Conclusions

The results of this study show that while TB "lay experts" knowledge seems fairly good, their perceptions suggest that stigma may play a significant role in case finding and case holding. Findings from this study are important in improving the societal supports to TB patients. It would seem, therefore, that community education should focus on improving attitudes and perceptions, thus potentially reducing stigma. It requires a patient centered approach, which starts with interventions targeting the intrapersonal level (empowerment, self help, advocacy and support group) to empower TB patients. The next step is involvement of TB patients in the development of stigma reduction programs at other levels [18]. TB patients' experiences are helpful at organizational/institutional and community level in developing training programs and new interventions that should contribute to stigma reduction rather than unintentionally enhance stigmatization. A shift of power relation and an active role of TB patients in this process could lead to more patient centered programs, empowerment of TB patients and stigma reduction. Furthermore, studying the actual situation in TB suspects and patients is necessary to confirm our study findings.

## Competing interests

The authors declare that they have no competing interests.

## Authors' contributions

All authors worked together on drafting the design for data gathering. VM of the Rhodes University of Grahamstown South Africa carried out the community study in collaboration with Trained Development Research Africa and local interviewers who conducted the interviews. JC and AN of the institute of Health Policy & Management (iBMG) The Netherlands performed the statistical analysis, and drafted the manuscript. VM and HF helped drafting the manuscript and contributed to refinement. All authors read and approved the final manuscript.

## Pre-publication history

The pre-publication history for this paper can be accessed here:

http://www.biomedcentral.com/1471-2458/10/72/prepub
